# Evaluating an interactive acceptance and commitment therapy (ACT) workshop delivered to trained therapists working with cancer patients in the United Kingdom: a mixed methods approach

**DOI:** 10.1186/s12885-022-09745-4

**Published:** 2022-06-13

**Authors:** Elisavet Moschopoulou, Debbie Brewin, Damien Ridge, Sheila Donovan, Stephanie J. C. Taylor, Liam Bourke, Gail Eva, Imran Khan, Trudie Chalder, on behalf of the SURECAN Grant Investigators, Kamaldeep Bhui, Liam Bourke, Trudie Chalder, Gail Eva, John Gribben, Ms Miriam Harris, Louise Jones, Ania Korszun, Paul Little, Paul McCrone, Adrienne Morgan, Damien Ridge, Rebecca Roylance, Stephanie J. C. Taylor, Mohamed Thaha, Peter White

**Affiliations:** 1 Wolfson Institute of Population Health, Barts and The London School of Medicine and Dentistry, Queen Mary University of London, London, UK; 2 Mind-Growth Mastery, Epsom, Surrey, KT19 0AA England; 3 College of Liberal Arts and Sciences, University of Westminster, London, UK; 4 Allied Health Professionals, Radiotherapy & Oncology, College of Health Wellbeing and Life Sciences, Sheffield Hallam University, Sheffield, UK; 5 Department of Sport and Health Sciences, Oxford Brookes University, Oxford, UK; 6 Department of Psychological Medicine, Institute of Psychiatry, Psychology and Neuroscience, King’s College London, London, UK

**Keywords:** Training, Acceptance and commitment therapy, cancer, Quality of life, Education

## Abstract

**Background:**

SURECAN (SUrvivors’ Rehabilitation Evaluation after CANcer) is a multi-phase study developing and evaluating an Acceptance and Commitment Therapy (ACT) intervention integrated with exercise and work when highly valued (thus we called the intervention ACT+), for people who have completed treatment for cancer but who have low quality of life. We developed a training programme for therapists working in different psychological services to be delivered over 2–3 days. Our aim was to evaluate the extent to which the training could improve therapists’ knowledge and confidence to deliver ACT+ to cancer patients in a trial setting.

**Methods:**

Three interactive workshops were delivered to 29 therapists from three clinical settings in London and in Sheffield. A mixed-methods approach was used. Questionnaires were designed to assess knowledge and confidence in using ACT+ with people who have low quality of life after cancer treatment. They were self-administered immediately prior to and after each workshop. Open text-based questions were used to elicit feedback about the workshops alongside a satisfaction scale. Semi-structured interviews were conducted with a purposive sample of therapists (*n* = 12) to explore their views about the training more deeply, and how it might be optimised.

**Results:**

Quantitative analysis showed that knowledge of ACT, as well as confidence in using the ACT+ intervention in this setting increased significantly after training (28.6 and 33.5% increase in the median score respectively). Qualitative analysis indicated that most therapists were satisfied with the content and structure of the programme, valued the rich resources provided and enjoyed the practice-based approach. Potential barriers/facilitators to participation in the trial and to the successful implementation of ACT+ were identified. For some therapists, delivering a manualised intervention, as well as supporting exercise- and work-related goals as non-specialists was seen as challenging. At the same time, therapists valued the opportunity to be involved in research, whilst training in a new therapy model.

**Conclusions:**

Training can effectively improve the knowledge and confidence of therapists from different clinical backgrounds to deliver a modified ACT intervention to cancer patients in a trial setting.

**Supplementary Information:**

The online version contains supplementary material available at 10.1186/s12885-022-09745-4.

## Background

According to Cancer Research UK, 50% of people diagnosed with cancer in England and Wales are predicted to survive 10 years or more, a statistic that has doubled in the last 40 years [1]. Although cancer survival varies widely worldwide, this favourable trend is in line with data from Europe and the United States where 5-year average survival rates have generally increased steadily over time and are now around 50 and 67% respectively [2, 3]. In the UK, about a third of patients living with and beyond cancer report poor quality of life (QoL), due to issues such as fatigue, the physical side effects of treatment, emotional distress including fear of cancer recurrence, and concerns about returning to work [4–6]. Although returning to work may help cancer patients regain a sense of normality [7, 8], evidence suggests that cancer survivors of working age have a higher risk of unemployment compared to healthy individuals [9, 10]. At the same time, keeping physically active can have a beneficial effect on the overall health and quality of life of cancer survivors and can help reduce side effects of treatment such as fatigue, anxiety and pain [11, 12].

Previous research from our group has shown that both a talking therapy such as cognitive behavioural therapy (CBT), and exercise-based interventions have some effect on improving the QoL of those living with and beyond cancer [13]. Since current interventions are only moderately effective, we set up the SURECAN study (Survivors’ Rehabilitation Evaluation after Cancer) to develop, pilot and evaluate a novel, person-centred psychological intervention based on Acceptance and Commitment Therapy (ACT). The potential for ACT to benefit cancer patients from different backgrounds and with varied degrees of difficulties and abilities relates to how it specifically constructs patient change. In contrast to traditional approaches to psychopathology, ACT is not concerned with categorising and treating patients’ symptoms. Instead, it focuses on establishing acceptance of what cannot be changed (e.g., cancer diagnosis, the risk of recurrence), while encouraging commitment to pursuing realistic change by prioritising personal values and encouraging psychological flexibility and adaptive behaviours.

Recently, studies have highlighted the broad applicability of ACT as a transdiagnostic intervention that can be delivered in different formats to meet the needs of different patient populations [14, 15]. The evidence base for ACT in long-term conditions was reviewed by Graham et al. [16] who reported that ACT has shown promise in improving several outcomes within a range of conditions (e.g. cancer, epilepsy, paediatric illness, multiple sclerosis, diabetes). Nevertheless, the authors emphasised the lack of methodologically rigorous studies. Empirical support for the application of ACT specifically in cancer has grown in recent years. Gonzalez-Fernandez & Fernández-Rodríguez (2019) [17] reviewed the evidence from studies of ACT in oncology settings and reported promising findings including improved patient quality of life and emotional wellbeing, as well as greater psychological flexibility. The authors concluded that large-scale randomised clinical trials (RCTs) comparing ACT with no treatment or with other efficacious therapies were warranted. In another recent review, Mathew et al. (2020) [18] examined 13 studies of ACT in different adult cancer survivors and concluded it is an effective intervention for common concerns such as anxiety, depression and fear of recurrence. The authors suggested that further research was needed to investigate the effects of ACT on other outcomes.

We developed an intervention based on ACT, to be integrated with exercise and work/meaningful occupation, in ways that are tailored to each participant’s personal values and goals. We called this intervention “ACT Plus (+)” to emphasise that therapists should bring up conversations in therapy around work and exercise, considering the evidence-base for them. If participants are interested in value-based goals related to exercise or work then the therapist will facilitate it. Equally, if participants do not hold values which include exercise or work then the focus of the therapeutic conversations will be around values and goals which are of personal importance. A theory-based and evidence-based approach was adopted to facilitate the iterative development of ACT+. Normalisation Process Theory [19] was used to guide this process and evidence from several different sources (i.e. qualitative work with patients and key stakeholders, evidence from the literature, as well as insights from patient and public consultations) was brought together to help us develop and refine the proposed intervention. NPT provides a framework within which to understand and evaluate key mechanisms that promote the successful implementation of new technologies and complex interventions [20]. In the SURECAN programme, NPT constructs (i.e. context, coherence, cognitive participation, collective action and reflective monitoring) are used as a sensitising tool to ask pertinent questions of patients and healthcare professionals, as well as in data analysis in order to robustly think through issues of intervention development, evaluation of delivery and trial issues. NPT was chosen as it is widely used in the UK [21], being consistent with Medical Research Council guidance for the development of complex interventions [22], including fulfilling a recognised need for strong theoretical foundations in intervention development and evaluation [19]. We also shaped the ACT+ intervention to be delivered in culturally sensitive ways and increase the likelihood that it would be acceptable to a culturally diverse population [23]. For example, we iteratively developed the ACT+ Participant Handbook, a resource for patients receiving ACT+, in consultation with patient and public representatives from diverse ethnic backgrounds (*N* = 8). They reviewed the handbook and commented on its cultural acceptability, language use and comprehension, as well as the appeal of the imagery.

ACT+ consists of eight weekly or biweekly sessions with a trained therapist using different modalities of delivery to suit individual needs (e.g., face-to-face, via telephone/video). Therapists delivering ACT+ sessions are supported by a therapy manual designed to provide guidance on what might be addressed during the course of therapy and to supply therapists with relevant prompt sheets and materials for the participants. The manual is intended to be a flexible guide to help therapists adopt a broader ACT-consistent approach. For example, although the ACT+ sessions follow a structure, therapists are encouraged to use a formulation-based approach and to tailor the proposed ACT+ session plans according to each participant’s needs and values. Similarly, the manual includes a variety of suggested metaphors, mindfulness exercises and worksheets. However, their use is not intended to be prescriptive and therapists are encouraged to use them as they see fit or to develop and use their own therapy aids.

In terms of who could deliver ACT+ in England, Improving Access to Psychological Therapies (IAPT), a National Health Service (England) initiative, recently widened its scope to provide therapy for patients with long-term physical health conditions who also report emotional distress, including those living with and beyond cancer [73]. Traditionally, they provide evidence-based psychological therapies for adults with depression and anxiety [24]. For the purposes of the trial, ACT+ could also be delivered by therapists in specialist secondary care services or by those in the charity sector whose responsibilities include supporting cancer patients.

This paper describes the development of an interactive multimodal training programme for therapists taking part in the SURECAN trial and its subsequent evaluation. Having developed the prototype ACT+, we a) developed a training programme to be delivered over 2–3 days to train therapists from different services in the structured ACT+ manualised intervention, and b) collected feedback from therapists who attended training to explore their experience and modify the programme accordingly. The aim of the evaluation was both to investigate the effectiveness of the workshop at improving therapists’ knowledge and confidence to deliver the manualised ACT+ intervention to cancer patients in a randomised clinical trial setting and to understand how we might improve the proposed training programme.

## Methods

### The training programme

Two experienced cognitive behavioural therapists developed iteratively an interactive, bespoke training programme in consultation with the wider SURECAN team. Both therapists had received extensive training in ACT. Three training workshops, each delivered over 2–3 days, were held in London and in Sheffield between November 2018 and January 2020. All workshops were held at university teaching rooms (i.e. Queen Mary University of London and Sheffield Hallam University). The location of each workshop was chosen based on trainees’ geographical convenience, however both trainees and trainers were reimbursed for any travel and accommodation costs incurred. Workshops were delivered between 9.45 am and 5 pm, with the third and final day finishing at 2 pm. A 15-minute break between sessions was taken in the morning and in the afternoon, while an hour-long break was allowed for lunch. Lunch and refreshments were provided throughout the workshop. The two therapists delivered all workshops, while members of the research team delivered specialist sessions. Our goal was to train therapists in the structured ACT+ clinical approach and to equip them with the confidence and knowledge to deliver the ACT+ manualised intervention, encouraging them to adopt a broader ACT-consistent approach towards working with cancer patients.

Workshops consisted of a range of didactic and experiential activities using PowerPoint and audio-visual skills demonstrations, role-plays, case discussions and group work. On the first and second day of the workshop, approximately 3.5 hours were devoted to PowerPoint presentation time, while about 2 hours were dedicated to group exercises and experiential activities. About 30 minutes at the end of each day were used to reflect on the content covered and to answer questions. Day 3 included less Power point time (about 1 hour) and more time was reserved for reflections, discussion and demonstrations guided by therapists’ needs. The training programme included topics such as insights into cancer diagnosis, teaching on the theory of ACT and techniques used in therapy (e.g. mindfulness, use of metaphors), the ACT+ model and structure of sessions, cultural sensitivity based on the findings from a qualitative SURECAN work-package [25], as well as supporting exercise- and work-related goals. Following therapists’ feedback after the first workshop, minor changes were made to the content. Information about the theory of ACT was expanded and the workshop was lengthened from 2 days to two and a half days to allow more time for final reflection. The second and third workshops were identical. A detailed description of the content covered in the training programme is provided in additional file 1. Therapists received the ACT+ Therapist Manual and the Participant Handbook, resources developed iteratively, specifically for SURECAN, in consultation with the study’s patient and public involvement group and community groups. Feedback from the study team and therapists who attended ACT+ training was also considered when developing further iterations of the resources.

### Participants

The opportunity to take part in the study and be trained was advertised in the NHS and third sector services interested in SURECAN. There were no financial incentives for therapists to participate in the trial and to attend the training workshop. Incentives to attend the workshop were primarily linked to therapists’ professional development (e.g. training in a new therapeutic model, being involved in research, gaining experience of working with this patient group). Therapists could attend the training programme free of charge.

Sixteen therapists across the three workshops were invited to take part in qualitative interviews and 12 agreed. Seven therapists from the first workshop, 4 from the second and 1 from the third workshop were interviewed A purposive sampling approach was adopted to seek the views of therapists working in different services, of different genders and with different qualifications. The sample comprised 7 therapists working in secondary care, 4 in IAPT services and 1 therapist in the charity sector. Three therapists were men. Six were clinical psychologists, 3 were CBT therapists, 2 were counselling psychologists and 1 was a hypnotherapist.

Therapists who attended the training workshops, as well as those who participated in qualitative interviews were not paid for their time as both activities took place during their working hours in agreement with their employer.

### Data collection

For the purposes of evaluating the training programme, all therapists were asked to complete a set of questionnaires upon their arrival to the training location prior to the start of the workshop (T1) and immediately after the end of the workshop (T2). A satisfaction questionnaire was distributed at the end of each workshop (T2). All survey responses were anonymised to ensure confidentiality. Semi-structured qualitative interviews [26] were conducted approximately 2 months after the workshop to elicit therapists’ thoughts on the workshop. Interviews lasted approximately 1 hour and were conducted face-to-face at therapists’ workplace or via telephone. Interview topics included therapists’ prior experiences of ACT, research and cancer care, their initial thoughts on the modified ACT+ model, the content and format of sessions, how the workshop might be modified and their confidence to deliver ACT+ (topic guide available as supplement – see additional file 1).

### Measures

In accordance with Kirkpatrick’s [27] training evaluation model, the impact of the workshop on therapists’ knowledge and confidence, as well as their satisfaction with the training, were assessed using self-administered questionnaires. In Kirkpatrick’s model, there are four consecutive levels (i.e. reaction, learning, behaviour, and results) each representing a more precise measure of training effectiveness. This model was chosen for its success in studies evaluating training for health professionals [28], and for its systematic approach. The questionnaires were designed by the study team to address key aspects of the workshop’s content and assess the first two levels of Kirkpatrick’s model; a satisfaction scale assessed “reaction”, while knowledge and confidence questionnaires assessed “learning”. The knowledge questionnaire was developed specifically for this study, whereas the confidence and satisfaction scales were based on instruments designed for a previous study [28].

Knowledge about living with and beyond cancer and the ACT model was assessed using a series of statements that participants categorised as “True” or “False (see Table 1). Each correct answer was given a score of 1 and a summated total score was calculated (maximum score = 10). Attendees were also asked about their confidence in delivering ACT+ using twelve questions scored on a five-point Likert scale (1 = Not Confident; 3 = Somewhat Confident; 5 = Completely Confident) (see Table 2). Finally, therapists were asked to rate their overall satisfaction with the quality of each day of the workshop using a scale from 1 (Needs Improvement) to 4 (Excellent). A mean satisfaction score for each day was calculated. Using open text-based questions, therapists were asked to identify (a) the best and (b) the most interesting features of the workshop, as well as to (c) suggest how the training might be improved.

**Table 1:** Percentage of correct responses to individual items on the general knowledge. Questionnaire (*N =* 29)^a^

Questionnaire Item	Correct answer	Percentage of correct responses	
		T1	T2
Q1) It is estimated that about a third of patients living with and beyond cancer report poor quality of life.	True	86.2	93.1
Q2) Quality of life is only affected by psychological problems.	False	93.1	89.7
Q3) People living with and beyond cancer are 40% more likely to be unemployed than those who have not had cancer.	True	79.3	100*
Q4) People who have recently finished treatment for cancer should avoid physical activity.	False	100	100
Q5) ACT tries to identify a problematic behaviour and reduce symptoms.	False	58.6	82.8*
Q6) In targeting unhelpful processes, ACT examines whether a thought is true or false.	False	82.8	100
Q7) ACT puts participants’ views about what they value most in their lives at the heart of the therapy.	True	100	100
Q8) The ACT therapeutic stance encourages the use of limited self-disclosure.	True	41.4	93.1***
Q9) There is substantial evidence ACT helps people who are living with and beyond cancer.	False	37.9	44.8
Q10) Values direct our behaviour and they can be cancelled out by a failure to achieve a goal.	False	72.4	75.9

^a^ McNemar’s test

* *p <* 0.05

** *p <* 0.01

*** *p <* 0.001

**Table 2:** Mean scores and standard deviations for each item on the confidence questionnaire (*N =* 29)^a^

Questionnaire Item	Mean (SD)	
	T1	T2
Q1) How confident do you feel about delivering ACT+ to participants living with and beyond cancer?	2.07 (0.96)	3.21 (0.82)***
Q2) How confident do you feel about structuring sessions based on the ACT+ therapeutic model?	1.97 (0.94)	3.14 (0.83) ***
Q3) How confident do you feel about detecting psychological inflexibility?	3.00 (0.89)	3.79 (0.73) ***
Q4) How confident do you feel about using the ACT therapeutic processes to improve psychological flexibility?	2.14 (0.79)	3.48 (0.74) ***
Q5) How confident to you feel about communicating the ACT model effectively using metaphors?	2.17 (1.04)	3.41 (0.73) ***
Q6) How confident do you feel about using mindfulness exercises in the context of the ACT model?	2.59 (1.15)	3.69 (0.71) ***
Q7) How confident do you feel about helping the participant to notice the stimuli (thoughts, feelings, situations, etc.) that hook them and take them away from the present moment.	3.28 (0.70)	3.72 (0.65)**
Q8) How confident do you feel exploring values and what is important to participants?	3.59 (0.78)	4.03 (0.57)**
C9) How confident do you feel about encouraging committed action?	2.97 (0.82)	3.76 (0.64)***
Q10) How confident do you feel about having a structured conversation with participants in order to identify actual or potential problems in the work place?	3.10 (0.82)	3.76 (0.69)***
Q11) How confident do you feel about giving participants living with and beyond cancer advice about how to develop exercise goals in context of ACT+?	2.34 (1.01)	3.59 (0.73)***
Q12) How confident do you feel about working with participants from a diverse ethnic background who may hold alternative health beliefs?	3.31 (0.85)	3.83 (0.71)**

^a^ Wilcoxon signed-ranks test

* *p <* 0.05

** *p <* 0.01

*** *p <* 0.001

### Analysis

Quantitative data were analysed using SPSS Version 25. Descriptive analysis showed that data were not normally distributed and therefore, non-parametric statistics were used. Wilcoxon signed-rank tests were used to compare knowledge and confidence scores before and after the training. McNemar’s tests and Wilcoxon signed-rank tests were used to compare responses to individual items of the knowledge and confidence questionnaires respectively at T1 and T2. Kruskal-Wallis tests were used to compare scores between more than two groups.

Analysis of qualitative data was conducted using content analysis for responses to open-ended questions and thematic analysis for interview data [29]. Interviews were digitally recorded and professionally transcribed verbatim. Transcripts were anonymised before input into NVivo 12, where coding reports were generated and analysis was undertaken. An inductive approach was adopted whereby after repeated reading of the transcripts to familiarise herself with the data, EM (who had been present at the workshops) coded the transcripts. The process of generating and refining the codes and coding the transcripts was documented by EM and discussed in team meetings with the qualitative lead (DR), the co-chief investigator (TC), who had delivered the training, and a co-researcher (SD) who was also familiar with the data, having conducted the interviews, as well as produced transcript summaries for discussion with the wider team. Qualitative findings, initially drafted by EM, were discussed and debated by all authors involved in the qualitative work, until agreement was reached on the findings.

### Ethical approval

This study formed part of developing and evaluating an educational course and therefore ethics approval was not required to administer the questionnaires. Ethical approval for the interviews was obtained by the Cornwall & Plymouth Research Ethics Committee (Reference number: 18/SW/0196).

## Results

### Quantitative results

In total, 29 therapists from three clinical settings took part in three workshops. Therapists in the first workshop in London were psychologists (*n* = 7) working in secondary care (i.e., Setting 1). Therapists in the second London-based workshop (*n* = 18) were CBT therapists working in IAPT services (i.e., Setting 2). Finally, participants in the third workshop (*n* = 4), which took place in Sheffield, were working in the charity sector specifically supporting patients living with and beyond cancer (i.e., Setting 3). 90% were women. Across all workshops, most participants were CBT therapists (44.8%) and clinical psychologists (31%) followed by counselling psychologists (17.2%) and hypnotherapists (6.9%). In the UK, CBT therapists are healthcare professionals who have been trained to a postgraduate level specifically to assess and support people with mental health problems using CBT. Counselling or clinical psychologists have usually trained to a doctoral level, they may specialise in different areas and may use a variety of therapies to treat people with a range of difficulties. The majority of therapists in the workshop (*n* = 22) had received some training in ACT prior to attending our workshop, although levels ranged from having had one lecture to having attended training over several days.

#### Knowledge

There was a statistically significant increase in therapists’ total knowledge score (Z = − 3.5, *p <* 0.001, *r* = 0.45) after the workshop (median = 9.0; interquartile range = 2.0) compared to before (median = 7.0; interquartile range = 3.0). Table 1 shows the correct response for each item of the knowledge questionnaire, as well as the percentage of accurate responses achieved pre- and post-training. 

A Kruskal-Wallis test was used to compare knowledge scores between the groups of therapists in the three different settings. There was a statistically significant difference in knowledge scores between groups before the training (χ^2^(2) = 9.12, *p* = 0.01) but only a trend effect afterwards (χ^2^(2) = 5.99, *p* = 0.05). Post hoc analyses with Bonferroni correction for multiple comparisons showed that secondary care psychologists scored significantly higher than charity-based therapists.

#### Confidence

A Wilcoxon signed-rank test showed a statistically significant increase in therapists’ total confidence scores between T1 (median = 32.5; interquartile range = 10) and T2 (median = 43.4; interquartile range = 6), Z = − 4.5, *p <* 0.001, *r* = 0.59. Table 2 shows item-by-item mean confidence scores at T1 and T2. All item scores increased significantly after the workshop.

There were no significant differences between the three clinical groups either before (χ^2^(2) = 3.81, *p* = 0.15) or after (χ^2^(2) = 3.06, *p* = 0.22) the workshop.

#### Satisfaction scale

Mean satisfaction rating for Day 1 was M = 3.00, (SD = 1.05) for Day 2 it was M = 3.14, (SD = 0.89), and for Day 3 it was M = 3.57, (SD = 0.60).

### Qualitative results

#### Satisfaction scale (open text-based questions)

The best features of the training as reported by participants included receiving useful resources and materials, the workshop and trainers’ approach (e.g., variety of training methods, experiential learning, knowledge), and learning about ACT and mindfulness. The most interesting features included learning about the core components of ACT and associated techniques, as well as learning about cultural influences and research processes.

In terms of improvements to the workshop, most therapists wanted more role-plays and demonstrations. Minor structural changes were also suggested (e.g., better signposting to materials, less content during day one). Attendees at the first workshop recommended content changes (e.g., further information on the theory of ACT).

Illustrative quotes are presented in Table 3.

**Table 3:** Therapists’ feedback about the ACT+ workshop through open text-based questions

Themes emerging from content analysis	Number of therapists citing a theme ^**a**^	Illustrative verbatim responses
**A. Best features of the workshop**		
Satisfaction with resources and materials provided during the workshop	14/28	“The materials – very thorough and user friendly” (T 10, W 2) “Excellent training packs/materials. The content is accessible and actually works in day-to-day life” (T 4, W 3)
The trainers’ approach and knowledge	15/28	“Knowledge, expertise, flexibility, approachability of all staff” (T 3, W 3)
Learning about the ACT model including new therapy techniques and skills and incorporating exercise- and work- related goals	11/28	“Mindfulness-based exercises, ACT model and seeing it in action, incorporating work and exercise using ACT+ approach” (T 4, W 2) “The simplicity of core themes. All the different strands – work and exercise” (T 4, W 3)
Experiential aspects of the training	11/28	“Good to have many different exercises and metaphors practiced together” (T 6, W 1) “Good mix of theory and practice” (T 4, W 3)
Satisfaction with the training set up	11/28	“Use of mixed models for learning –role plays, videos, lecture notes, quizzes” (T 5, W 2) “Variety of presenters” (T 3, W 2)
**B. Most interesting/ new things learned**		
Learning about the theory of ACT, and about techniques and skills used to deliver ACT	24/28	“Some new metaphors/exercises to help patients” (T 7, W 1) “Stuck loops is a very useful concept to address problems with internal dialogue” (T 3, W 2) “How to identify values and encourage clients to live by them” (T 8, W 2) “Learning to be more aware and accepting of emotions and thoughts” (T 10, W 2) “Value of being able to “sit” with difficult thoughts and feelings, accept them rather than avoiding, fighting or trying to get rid of, and still move forward with life” (T 2, W 3) “Working with experiential avoidance” (T14, W2)
Learning about research	4/28	“Insight into process of RCT/training therapists for research” (T1, W1) “Learning about the research and evidence for 1) ACT 2) cancer 3) cultural sensitivity 4) exercise 5) employment issues” (T5, W2)
Learning about the role of culture in therapy, as well as how to support exercise- and work-related goals	5/28	“Different ways to adapt to the client’s own knowledge/practices (e.g. Islam – supplications) and language/words” (T2, W2) “Looking and becoming more aware about language in communication” (T15, W2) “Getting involved in discussion about work (previously avoided)” (T14, W2) “Talking about exercise in session” (T16, W2)
**C. How the training might be improved**		
More practise, role-plays and demonstrations	11/28	“Could do more practice from the start and maybe some more demonstrations/role-plays” (T13, W2) “Great training, maybe more live role-plays observed, fishbowls for use to ask ACT-consistent questions or be involved” (T5, W2)
Structural changes	9/28	“More obvious signposting during the power point” (T3, W1) “The first day was very intensive and overwhelming. Breaking it down with more role-plays or videos could help in the future” (T8, W2) “Finish the sessions at 4 pm, as there is so much material/learning” (T4, W3) “Having more info in advance, e.g. the manual might have helped.” (T4, W1)
Content changes	7/28	“A re-cap/overview of ACT would have been helpful to orientate us.” (T4, W1) “More background on ACT core processes” (T7, W1)

^*a*^ *Data from one attendee was missing therefore we collected feedback from 28 therapists in total*

*T = Therapist; W = Workshop*

#### Interviews

Twelve therapists were interviewed. Three themes, each with subthemes (as numbered in brackets below), emerged from our thematic analysis: 1) therapist background and stance towards ACT, 2) content and structure of the workshop, and 3) workshop outcomes. Supporting quotes are presented in Table 4.

**Table 4:** Qualitative themes and subthemes from interviews with therapists who attended the ACT+ workshops

Theme	Subtheme	Example quote
**1. Therapist background and stance towards ACT**	1.1 Therapists’ stance towards ACT (in the context of current practice)	I think ACT as a model, […] it’s very much about us coming alongside a patient rather than us being experts. […] it’s not that something’s broken in the patient that needs to be fixed. It’s really that they’re making understandable changes that, in the circumstances they’ve been in, but now we need to look at well, what’s working and could you do something different? So I really like that it’s not blaming, it’s not punitive and it’s very much about what are we going to do about how you’re managing in the here and now. (W1–2) I think it’s pertinent to clients, I think it could be useful. It moves away from some of the traditional CBT that seems less helpful to people with physical health conditions, particularly in this instance, cancer. Because it does tap into who that person is, and not who they used to be, but still who they are. And allows you to then move forward with that, rather than keep on going back to the past. (W2–4)
	1.2 Delivering a manualised intervention	[…] having the amount of experience that I have […], I think the risk is engaging in this trial might not make enough use of that level of experience and flexibility and adaptability. […] as an intervention it may be an intervention that’s better suited to be provided by people with a lower level of experience. Because it provides a scaffolding for their work, which would be helpful, rather than constraining. (W1–6) So doing a trial where you just have to do something in particular really quite appeals to me, and I just see how that is. But […] if I feel like something else might be helpful, then I’m not allowed to do that, I’ve just got to do what I’m doing within the model, then I guess that will be a bit of a dilemma. Well, not a dilemma, but that will be maybe, yeah, challenging. But I guess, yeah, we’ve got to try these things and see if they work and see if they have value in that sense. (W2–3)
	1.3 Supporting work- and exercise-related goals	We have like a local exercise on prescription scheme, and we’ve got a member of the team whose, that’s kind of their thing. And so it’s quite prominent, and equally the employment obviously, is IAPT, the employment support workers that we have here. […]So it feels in line with what we’re already doing. I suppose the difference for me is the sense of me doing all of those things. (W2–3) […]But yeah, how much are we expected to do? To get into that part of it. And how much does a client want you to do that? Because they’re coming to you for therapy. So probably the expectation might not be I’m coming to you so you can sort out my issues with my employer, and talk to me about the Disability Discrimination Act and what that means for me and how I can fight my corner. But again, we’d bring that back to problem-solving. (W2–4)
**2. Content and structure of the training workshop**	2.1 Training content	Maybe at least one other day covering ACT in itself would’ve been really helpful. Because everybody came with different levels of knowledge about ACT. […]So a separate component teaching that, before moving on to how it would work in practice in six sessions for this particular patient group would’ve been really useful. (W1–1) But the SURECAN training was good for me because it was pitched at that level of people who didn’t know so much about ACT already sort of thing. […] I didn’t come out of it feeling like oh, there were things that we didn’t cover. (W2–3) It was a nice, flexible approach. Everything … the pace was great. We knew what we were doing at the end of each session. So I think the length of time devoted to each subject and the mix of role-play and theory. Yeah, I couldn’t say do anything different […] we all thought it was great, we really enjoyed it. Yeah, I don’t think there was anything, you could do anything different there. (W3–1)
	2.2 ACT+ materials and resources	[…] there were lots of worksheets, both for clinicians but also for patients to go through. And I think that’ll be great for them to have that information. I know in the service I work in we don’t have access to anything like that. So it’s always oh, I’ll print you this off, or there’s a bit of paper here. Whereas when they go to see IAPT they get a really nice glossy brochure. And I do think, I think it gives a really strong message to people that people have really invested in trying to deliver something helpful to them. (W1–2) I think holding onto that manual is useful with the ACT+, with the SURECAN, but also generally speaking I think I can probably use that in other areas of my practice. A nice little manual to have. (W2–4)
	2.3 Training format	I seem to remember that there was a reasonable mix in that there was a fair amount of presentation. But there was some role-play and there was also some discussion time. (W1–5) I think the training was very, very well designed. I was very impressed with it. […] This training did give me a lot of confidence. I loved the way that it was delivered. The venue was amazing. The trainers were very accommodating, they were very open and understanding and inviting. The people were really nice, but I think that’s just coincidental. […] Everyone felt very comfortable speaking about things. There wasn’t really a person who didn’t speak […] (W2–2)
**3. Workshop outcomes**	3.1 Knowledge and confidence	I think it was useful in breaking down a few prejudice and preconceptions. Maybe my expectations were too low in terms of people getting back to the work that they were doing and … (W2–1) […] Whereas I thought with this, actually I’m learning a skill, like the ACT+ that’s quite specific to the cancer survivors. And actually then it doesn’t stop there because I’m going on in a way to be assessed. So I might be able to hone those skills better, and actually be able to deliver, hopefully, quite a good therapy programme. (W2–4)
	3.2 Engagement and commitment	Because I remember every one of us, when we went for a lunch break, was saying oh gosh, I hope I end up seeing a client. […] And I thought to myself oh gosh, I hope I will be given a client because this sounds really exciting, it sounds like a good training. I want to train and get on with it. (W2–2) So with a trial you’re looking to find out what works, what works best, what doesn’t work. So I’m very open to it, and actually quite excited by it. And looking forward to learning from it. (W3–1)

##### Theme 1: therapist background and stance towards ACT

The majority of therapists reported that the ACT+ model fitted well with their current practice (1.1). Despite varied levels of experience of using this approach, therapists across all settings commented positively on the applicability of ACT in cancer care. Many highlighted the appeal of the ACT therapeutic stance, while others valued the notion of psychological flexibility.

Depending on experience and clinical role, therapists expressed different views about taking part in a research trial and the intervention itself. Psychologists were curious but also sceptical about delivering a manualised intervention (1.2). The manualised approach was considered constraining by experienced clinicians who valued their ability to adapt therapy drawing from different therapeutic approaches. Nevertheless, other views were expressed by the same individuals (‘very motivated to do it, very open to being trained’). Some participants mentioned that this adaptive approach to therapy which seemed integral to their practice might impede adherence to the standardised approach required in a clinical trial setting. Perceptions of constraint were linked to therapists’ prior training. Thus, for some IAPT therapists, who primarily offer structured CBT, ACT was perceived as allowing comparatively greater freedom in the use of clinical skills than reported by psychologists. However, IAPT therapists did report anxieties about deviating from the intervention protocol.

Participants perceived exercise and meaningful occupation as “important” domains that “lend themselves really well to a values-based conversation in ACT”. However, supporting work- and exercise-related goals as non-specialists in the context of ACT+ was also perceived as challenging. Therapists across clinical settings described their routine practice as having “basic conversations” around physical activity/ work and referring patients to specialist colleagues.

##### Theme 2: content and structure of the training

Most therapists appeared satisfied with the training programme in terms of the topics covered (2.1), the materials provided (2.2), and the format (e.g., duration, pace, various teaching modalities to suit different learning styles, variety of presenters) (2.3). Some of the therapists who attended the first workshop recommended adapting the content and duration of the workshop in order to dedicate more time to the fundamentals of ACT and the underpinning theory, introducing a standard knowledge base, irrespective of individuals’ prior familiarity with ACT. The training programme was modified accordingly and therapists who attended subsequent workshops reported the approach was flexible enough to meet therapists’ individual needs. Some therapists suggested that the lived experience of cancer could be covered in greater detail. Several therapists had never worked in cancer care therefore their knowledge of cancer patients’ experiences was limited.

Therapists enjoyed the experiential and interactive aspects of learning (e.g., mindfulness exercises, group activities) and found role-plays particularly helpful. Attendees also spoke highly of the expertise and “warmth and enthusiasm of the trainers” who were seen as having created an enjoyable learning environment.

##### Theme 3: training outcomes

Therapists reported that the training had equipped them with the knowledge needed to deliver ACT+ (3.1). Participants gained greater confidence in their abilities, and with some preparation and practice, believed they would be able to deliver the intervention adequately. For those without any prior experience of ACT, the ACT+ Therapist Manual was seen as “something that will give them confidence” going forward. Participants stated that they gained knowledge of strategies commonly used in ACT. Additionally, the workshop offered a framework within which to practice ACT outside the context of the trial. For some, taking part in the study was an opportunity to get involved in research or to work with a different client group, whilst also training in a new therapy model. Overall, participants registered enthusiasm about their trial involvement (3.2) and were keen to begin.

## Discussion

We developed a bespoke training programme for therapists working in different psychological services to be delivered over 2–3 days. Our aim was to train therapists in the structured ACT+ therapy manual and to evaluate whether training could increase therapist knowledge of ACT, as well as their confidence to deliver the ACT+ intervention to patients living with and beyond cancer, in the context of the SURECAN randomised controlled trial.

Our quantitative results showed that training was well-received and resulted in improved outcomes as reported by trainees themselves. Although pre-training total scores were relatively high, therapists’ knowledge increased significantly after the workshops with the greatest improvements seen on items related to key aspects of ACT (i.e. items 5 and 8 of the knowledge questionnaire focusing on the aims of ACT and the therapeutic stance respectively). Inspection of average scores for the three groups of therapists working in different settings showed minor differences in knowledge scores pre-training but no differences after the training. The small sample size precludes us from treating subgroup differences as significant effects but any discrepancies might reflect therapists’ prior education and training. Most therapists in our sample had received some training in ACT prior to the workshop.

After the workshops, therapists reported significantly higher confidence in their abilities to deliver the ACT+ intervention and to manage common difficulties in the cancer survivorship phase. Confidence levels did not differ between therapist groups either before or after the training.

Similarly, qualitative analysis showed that participants gained knowledge of ACT; they enjoyed the practice-based nature of the workshops; and were satisfied with the resources provided. Training was effective in upskilling therapists taking part as some were able to proactively apply new learnings from the workshop to their wider practice ahead of the start of the trial. Participants favoured more demonstrations and skills practice, however that would require a longer training programme. Feedback from therapists who attended the first workshop was acted upon and resulted in iterative changes to the content and duration of the workshop, which led to improvements for subsequent trainees. Nevertheless, the integration of work and exercise support alongside ACT continued to create concerns for some of the therapists around specialist expertise. Finally, subgroup differences were identified in qualitative analysis too. These were largely pertaining to the manualised nature of the ACT+ intervention with clinical psychologist and IAPT therapist interpretations of the ‘flexibility’ in the approach diverging.

Our findings echo previous evidence primarily from the CBT literature suggesting that brief interactive workshops are an effective means of knowledge acquisition, at least in the short-term [28, 30–32]. Our workshop effectively improved the knowledge and confidence of therapists across different disciplinary groups, with varied levels of experience of cancer care who work in different clinical settings. Potential barriers to participation in the trial or to the successful future implementation of ACT+ include using a manualised approach which some therapists perceived as constraining, as well as the integration of exercise and work support. Seeing as therapists were generally in favour of the integration of work and exercise support consistent with values, any concerns around their ability to provide adequate support are hypothesised to reflect a lack of confidence and/or knowledge with regards to addressing these topics. Furthermore, being trained to deliver a specific manualised approach in the context of a trial may have naturally increased therapists’ anxieties about any deviations from the manual. Nevertheless, therapists’ lack of confidence might also be linked to contextual factors (e.g., current service configuration, therapist professional background and training). Any such differences amongst therapists will be taken into account when considering how we can better address their training needs in the future. Concerns over supporting work-related goals are important to try and address as evidence suggests that health professionals’ beliefs about their ability to address work-related issues leads to the topic never being raised which in turn leads to cancer patients not being supported [10]. Rethinking how to get across key messages on supporting exercise and work-related goals as non-specialists might enable us to reduce therapists’ anxieties in future workshops. Therapists delivering ACT+ in the context of the SURECAN trial will be offered clinical supervision designed to contribute towards maintaining training gains. Therefore this might be one way to support them and build their confidence around any specific issues of concern.

A strength of our study is the multi-modal measurement approach taken. We used both qualitative and quantitative methods to obtain rich data. However, the lack of multiple independent coders in qualitative data analysis should be noted as a study limitation. Another strength is the high evaluation response rate across the three workshops. The study also benefits from the use of Kirkpatrick’s model which allows for the systematic evaluation of training and has been previously used in similar studies [28]. However only the first two levels of the model (reaction and learning) were assessed. A limitation of the study is the use of self-report study-specific questionnaires. Also, administering the questionnaires at the training location may have increased the likelihood of introducing positive response bias due to knowing the trainers would see the results, albeit anonymised. Another limitation is the lack of objective assessment of participants’ clinical skills using ACT pre- and post-workshop. Therefore, an important addition could be to evaluate long-term skill acquisition and maintenance in accordance with Kirkpatrick’s model. ACT+ sessions will be recorded and rated for treatment fidelity enabling us to assess skill implementation during the planned trial.

## Conclusions

In conclusion, our study has shown that a brief interactive training workshop can improve the knowledge and confidence of therapists from different clinical backgrounds to deliver a modified ACT intervention to cancer patients. Taking into account the emerging evidence base for the use of ACT in cancer [15, 17, 18] and other patients groups [14, 16], training healthcare practitioners to incorporate ACT into their supportive care practice is likely to be useful. We believe that our training programme can be a basis for developing and delivering effective ACT training to a variety of healthcare professionals who provide psychological support to those living with and beyond cancer and those with other long-term conditions. Further studies are needed to investigate the specific components of training that may have an impact on clinical skills and consequently on patient outcomes.

## Supplementary Information

**Additional file 1.** Content of the ACT+ Training Programme and Topic Guide for Therapist Interviews post ACT+ training. Description of data: Two tables are included in this word document. S1 presents in detail the content and structure of the ACT+ training programme. S2 presents the topic guide used for the purposes of the qualitative interviews reported in this article.

## Data Availability

The datasets used and/or analysed during the current study are available from the corresponding author on reasonable request.

## Abbreviations

ACT: Acceptance and Commitment Therapy
ACT+: Acceptance and Commitment Therapy Plus
CBT: Cognitive Behavioural Therapy
IAPT: Improving Access to Psychological Therapies
M: Mean
RCT: Randomised Clinical Trial
SD: Standard Deviation
SPSS: Statistical Product and Service Solutions
SURECAN: SUrvivors’ Rehabilitation Evaluation after CANcer
QoL: Quality of Life
