# Supplementary material for: Evaluating an interactive acceptance and commitment therapy (ACT) workshop delivered to trained therapists working with cancer patients in the United Kingdom: a mixed methods approach
Source: BMC Cancer. 2022 Jun 13;22:651. doi: 10.1186/s12885-022-09745-4 (PMC9195438; doi:10.1186/s12885-022-09745-4)
Supplement: Supplementary file 1 — Additional file 1. Content of the ACT+ Training Programme and Topic Guide for Therapist Interviews post ACT+ training. Description of data: Two tables are included in this word document. S1 presents in detail the content and structure of the ACT+ training programme. S2 presents the topic guide used for the purposes of the qualitative interviews reported in this article. [file 12885_2022_9745_MOESM1_ESM.docx]

**Evaluating an interactive acceptance and commitment therapy (ACT) workshop delivered to trained therapists working with cancer patients in the United Kingdom: a mixed methods approach**

**Supplementary** **file**

| **S1 - Content and structure of ACT+ Training Programme** | | |
| --- | --- | --- |
| **Workshop session** | **Content** | **Practical exercises /experiential work** |
| DAY 1 | | |
| Background to the SURECAN Study | Cancer as a long term condition: prevalence, risk, routes to diagnosis, treatments | A mindful introduction – come into the present using your five senses |
|  | Recognition of importance of psychological support; common needs of people living with and beyond cancer |  |
|  | Current interventions |  |
| The SURECAN programme | Rationale, aims and programme of research |  |
| Introduction to ACT | Relational Frame Theory |  |
|  | The ACT Model – core psychological processes and psychological flexibility |  |
|  | The ACT Hexaflex |  |
| The ACT+ Model and Framework | Open-Aware-Active: the “Three Pillars”, a simplified version of the ACT Hexaflex; workability; the ACT therapeutic stance; use of metaphors; mindfulness | Experiential exercise: “ACT in a Nutshell”  “Two mountains” metaphor and alternative ways to explain the ACT therapeutic stance  “Passengers on the Bus” metaphor |
| ACT+ Sessions and the ACT+ Therapist Manual | Overview of sessions and stages of therapy; Overview of therapy resources (Participant Handbook and Therapist Manual) | Exercise: practice using mindfulness |
|  | Structuring sessions: typical content and tips | Video clip on structuring sessions, reflection and skills practice  Experiential exercise: introduce a mindfulness exercise |
| Engagement, Assessment & Formulation | Engagement in the first sessions and introducing ACT | Exercise:  Using the What Works Plan for yourself |
| DAY 2 | | |
| Exploring values & linking to goals and actions | Exploring our values; values and goals; competing and conflicting values; agreeing tasks between sessions | Exercise: using “values cards” in pairs  Exercise: Agreeing between session tasks and using Weekly Committed Action sheets  Choice point video clip |
| Skills for overcoming ‘Stuck Loops’ and increasing flexibility | Identifying “Stuck Loops”; techniques to address stuck loops | Case studies: Review barriers to action  Metaphor: The unhelpful repetitive Parrot; Aircraft oxygen mask analogy;  Mindfulness Exercise: The compassionate mind  Mindfulness exercise: Thought Train |
| Ending therapy and building on progress | Validating and building on progress; How to maintain flexibility and strengthen self-care looking to the future; resources and support; dealing with set backs | Exercise: practise putting it all together and maintaining momentum |
| How to integrate exercise, if part of participant's  value-based goals | What do we mean by exercise and current guidelines for cancer patients; Key exercise metrics; Reviewing progression; Maximising adherence; Setting up smart exercise goals; Accessing exercise resources in the community | Pop quiz: test your knowledge of the body  Case study |
| How to integrate work-related conversations, if part of participant's  value-based goals | Evidence on work and cancer; values vs stressors; the characteristics of “good work”; Risk factors for work problems; common issues; reasonable adjustments and sources of support at work; having a structured conversation to identify work issues and set goals | Case studies |
| DAY 3 | | |
| ACT+: Recap and further practice | Recap of the ACT approach and theory; Pitfalls in ACT; Q&A | Skills role plays and demonstrations |
| Cultural considerations | Findings from our meta-ethnography of cultural influences on psychological interventions and translations into the work of therapists | Role playing /demonstrations at therapists’ request  Exercise: reflect on values and goals – choose one value you hold dear |
| Troubleshooting – Next steps and reflections on the training | Open discussion and reflections on the training; trial logistics and next steps |  |

| **S2** |
| --- |
| **Topic Guide for Therapist Interviews post ACT+ training** |
| 1. Did you have any experiences prior to training that might have influenced you?  - Prompt: How open were you to hear something new? / Or maybe it wasn’t new? |
| 1. What experience have you had of delivering therapy in context of trial, if any?  - Prompt: EG RE being closely scrutinised / receiving supervision / having therapy rated? |
| 1. How comfortable do you feel working on a trial, where the intervention may or may not work? |
| 1. What were your initial thoughts about ACT+?  - Prompts: Does it make sense to you? Prior experience of ACT? |
| 1. Elicit thoughts on the ‘physical activity’ component. |
| 1. Elicit thoughts on the ‘meaningful occupation’ component. |
| 1. Was the length of time allocated for the ACT+ training appropriate? |
| 1. Was the pace and format of the training sessions appropriate?  - Prompt: Was there a sufficient mix of presentations, interactive/role play sessions, and discussion time? |
| 1. Were the training sessions flexible enough to meet different individuals’ needs? |
| 1. Do you anticipate any difficulty in participants (*patients /individuals receiving the therapy*) using the manual? |
| 1. How could the manual/s be modified? (*therapist and/or participant manual*)  - Prompt: Anything that worked well, or didn’t work well? |
| 1. Would you feel confident to deliver ACT+ in the pre-pilot study? |
| 1. How could the training be modified?  - Prompt: Anything that worked well, or didn’t work well? |
| 1. How does ACT+ (the intervention therapy) fit with you as a person / your natural style of being? |
| 1. Anything else you would like to say? |
